# Beyond radiation dosimetry: metabolic burden and a possible healthy worker survivor pattern in the medical imaging workforce

**DOI:** 10.3389/fpubh.2026.1819933

**Published:** 2026-06-17

**Authors:** Saike Yan, Jianhui Wu, Yuanping Liu, Wen Si, Shengguang Yan

**Affiliations:** 1 North China University of Science and Technology College of Public Health, Tangshan, Hebei, China; 2 Tangshan Gongren Hospital, Tangshan, Hebei, China

**Keywords:** healthy worker survivor effect, metabolic dosimetry, NAFLD, occupational maladaptation, radiology workforce

## Abstract

**Objective:**

Current occupational health protocols in radiology remain strongly focused on ionizing radiation while underemphasizing metabolic health concerns potentially associated with sedentary and shift-based occupational environments. This study examined the prevalence of Non-Alcoholic Fatty Liver Disease (NAFLD) in medical imaging professionals and evaluated whether the observed tenure-related pattern was more consistent with early-career occupational burden and possible survivor-selection processes than with a simple linear cumulative-risk model.

**Methods:**

A cross-sectional analysis was conducted among 235 medical imaging professionals. NAFLD was diagnosed by abdominal ultrasonography according to APASL-based criteria. Restricted Cubic Spline (RCS) regression and multivariable logistic models were used to evaluate tenure-related patterns and metabolic predictors. Supplementary analyses further assessed waist circumference, work-schedule characteristics, multicollinearity using variance inflation factors (VIFs), and model robustness across alternative specifications.

**Results:**

The cohort exhibited a NAFLD prevalence of 47.7%. Occupational tenure showed a non-linear pattern, with prevalence rates of 44.1%, 61.9%, 32.5%, and 52.3% across the <5 years, 5–10 years, 10–20 years, and >20 years groups, respectively. Participants with NAFLD had a larger waist circumference than those without NAFLD (112.7 ± 5.1 cm vs. 105.6 ± 6.5 cm). Across sensitivity analyses, BMI remained the most stable metabolic predictor (OR 1.45–1.49), whereas the tenure-related effect was less stable. These findings are compatible with a possible survivor-selection pattern but require cautious interpretation.

**Conclusion:**

These findings do not support a simple linear interpretation of tenure-related metabolic risk. Instead, they suggest that adiposity-related metabolic burden is more robustly associated with NAFLD than occupational tenure alone, and that the observed tenure pattern may be compatible with survivor-selection processes. Future studies should directly quantify shift-related, circadian, and behavioral exposures, and metabolic surveillance may usefully complement existing dosimetry-centered occupational monitoring.

## 1 Introduction

Contemporary diagnostic radiology presents a distinct occupational disparity. While radiologists serve as the primary diagnosticians for metabolic dysfunction-associated steatotic liver disease (MASLD) and metabolic syndrome (MetS), the workforce itself is exposed to significant, yet unquantified, metabolic risks (1, 2). Although the profession strictly enforces the “As Low As Reasonably Achievable” (ALARA) principle for ionizing radiation—a hazard that has been effectively minimized through modern engineering controls—current surveillance protocols fail to address the metabolic burden driven by the transition to high-volume, sedentary, and shift-based workflows (3–5).

Emerging data indicate that diagnostic radiologists sustain sedentary periods exceeding 8 h daily, an inactivity burden often surpassing that of administrative personnel (6). This prolonged immobility attenuates non-exercise activity thermogenesis (NEAT) and downregulates lipoprotein lipase expression, establishing a pro-inflammatory metabolic milieu that remains refractory to recreational exercise (7). Concurrently, circadian misalignment driven by shift work—characterized as “chronodisruption”—has been implicated in the pathogenesis of insulin resistance and central adiposity, independent of positive energy balance (8, 9). However, although shift work, circadian disruption, and prolonged sedentary behavior provide biologically plausible contextual mechanisms for metabolic risk in this workforce, these exposures were not directly quantified using validated behavioral instruments in the present baseline dataset and are therefore interpreted here as contextual factors rather than directly measured causal drivers.

Notwithstanding these documented risks, occupational health surveillance remains entrenched in radiation dosimetry. A distinct surveillance asymmetry persists: while ionizing radiation exposure is subject to mandatory longitudinal quantification, there exists no equivalent ‘metabolic dosimetry' to monitor the cumulative burden of circadian misalignment or visceral adiposity (10). Consequently, historical epidemiological models have frequently been predicated on a linear dose-response assumption regarding occupational tenure, thereby neglecting the “Healthy Worker Survivor Effect” (HWSE)—a selection bias wherein metabolically susceptible individuals are selectively censored from the workforce (11).

This study characterizes the prevalence of NAFLD in the medical imaging workforce through a cross-sectional analysis. Specifically, the objectives were to examine whether the observed tenure-related pattern is more consistent with a non-linear early-career burden and possible survivor-selection process than with a simple linear cumulative-risk model, and to distinguish metabolic predictors from occupational duration. Although MASLD reflects current clinical nomenclature, the present cohort outcome was operationalized using an ultrasound-based definition and exclusion framework historically aligned with NAFLD terminology. Accordingly, we retain “NAFLD” when referring to the study-defined endpoint while acknowledging the broader relevance of these findings to the contemporary MASLD framework.

## 2 Methods

### 2.1 Study design and population

This study was conducted as a cross-sectional analysis of baseline data from an occupational health cohort established in 2023. The primary analytic cohort for the present manuscript consisted of 235 medical imaging professionals employed within a regional hospital network. The cohort included radiologists, technicians, and a small number of nurses. In addition to demographic and occupational tenure data, occupational category and work-schedule characteristics were summarized where available to further characterize heterogeneity across career stages.

To strictly isolate metabolic determinants from other etiologies of liver disease, the following exclusion criteria were applied: (1) positive serology for viral hepatitis B or C; (2) significant alcohol consumption (defined as >210 g/week for men and >140 g/week for women); (3) use of hepatotoxic medications or corticosteroids within the past 6 months; and (4) incomplete data regarding occupational tenure. The final analytic cohort consisted of 112 subjects diagnosed with NAFLD and 123 non-NAFLD controls.

### 2.2 Ethical considerations

The study protocol adhered to the ethical guidelines of the 1975 Declaration of Helsinki and was approved by the Institutional Review Board (IRB) of the participating hospital. Written informed consent was obtained from all participants prior to data collection.

### 2.3 Anthropometric and biochemical measurements

Data regarding demographic characteristics and occupational history, specifically the duration of radiation work (years), were obtained through structured interviews and verified against human resource records. Anthropometric measurements, including height, weight, and blood pressure, were performed by trained personnel. Body Mass Index (BMI) was calculated as weight in kilograms divided by the square of height in meters (kg/m^2^). Waist circumference was additionally evaluated in supplementary analyses as a marker of central adiposity. Work-schedule descriptors, including shift type, monthly night-shift frequency, and years of night-shift work, were also summarized where available.

Venous blood samples were collected after an overnight fast of at least 8 h. Serum biochemical parameters, including Alanine Aminotransferase (ALT), Aspartate Aminotransferase (AST), and Uric Acid (UA), were analyzed using standard automated enzymatic methods at the central clinical laboratory.

### 2.4 Diagnostic criteria

The diagnosis of NAFLD was established based on abdominal ultrasonography performed by experienced sonographers who were blinded to the participants' clinical and occupational status. Consistent with the guidelines of the Asian Pacific Association for the Study of the Liver (APASL), NAFLD was defined by the presence of characteristic sonographic features—specifically, diffuse increase in liver echogenicity (“bright liver”), vascular blurring, and deep beam attenuation—in the absence of secondary causes of hepatic steatosis.

### 2.5 Statistical analysis

Continuous variables are presented as mean ± standard deviation (SD) and were compared using the independent Student's *t*-test. Categorical variables are expressed as frequencies (percentages) and were compared using the χ2 test. Univariate and multivariate logistic regression analyses were performed to identify independent risk factors for NAFLD. Variables yielding a *P*-value <0.10 in the univariate analysis were entered into the multivariate model. To evaluate non-linearity in the association between occupational tenure and NAFLD risk, we employed Restricted Cubic Spline (RCS) regression adjusted for Age, BMI, and Uric Acid, with three knots located at the 10th, 50th, and 90th percentiles. To further assess robustness, supplementary sensitivity analyses were performed using four logistic model specifications: Model A (Age + BMI + Uric Acid), Model B (Work Years + BMI + Uric Acid), Model C (Age + Work Years + BMI + Uric Acid), and Model D (Age + Work Years + BMI + Uric Acid + Sex + Occupational Category). Additional models evaluated waist circumference and shift type. Multicollinearity was assessed using Pearson correlation coefficients and variance inflation factors (VIFs). Missingness of supplementary variables was summarized descriptively.

## 3 Results

### 3.1 Baseline characteristics and metabolic burden

**Figure 1:**
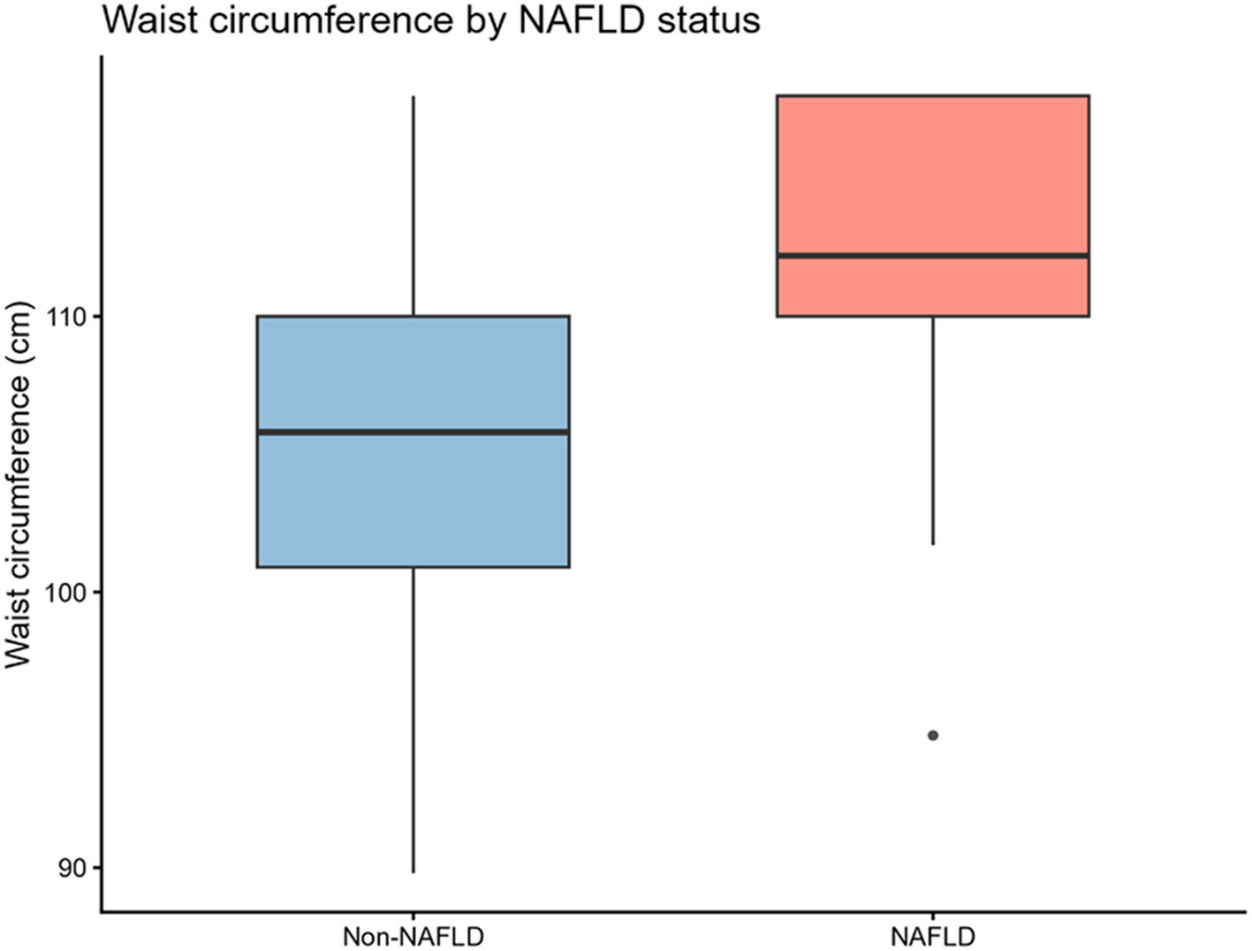
Waist circumference by NAFLD status. The boxplot shows a right-shifted waist circumference distribution in the NAFLD group, consistent with greater central adiposity among affected participants.

The analytic cohort comprised 235 medical imaging professionals, yielding an overall NAFLD prevalence of 47.7% (112/235). Comparative analysis demonstrated that the NAFLD subgroup was significantly older (40.3 ± 10.4 vs. 35.4 ± 9.1 years) and had a higher BMI (27.0 ± 4.3 vs. 22.5 ± 3.4 kg/m^2^) than the non-NAFLD subgroup. Biochemically, the NAFLD subgroup also showed higher uric acid concentrations (388.5 ± 99.1 vs. 317.9 ± 93.1 μmol/L). Supplementary analyses further showed that participants with NAFLD had a larger waist circumference than those without NAFLD (112.7 ± 5.1 cm vs. 105.6 ± 6.5 cm) (Figure 1). Waist circumference was moderately correlated with BMI (r = 0.556), and in a waist-based model adjusted for age and uric acid, waist circumference remained significantly associated with NAFLD (OR 1.19, 95% CI 1.12–1.27, *P* = 1.41 × 10^−8^), reinforcing the importance of central adiposity in this cohort.

### 3.2 Non-linear prevalence across occupational tenure

**Figure 2:**
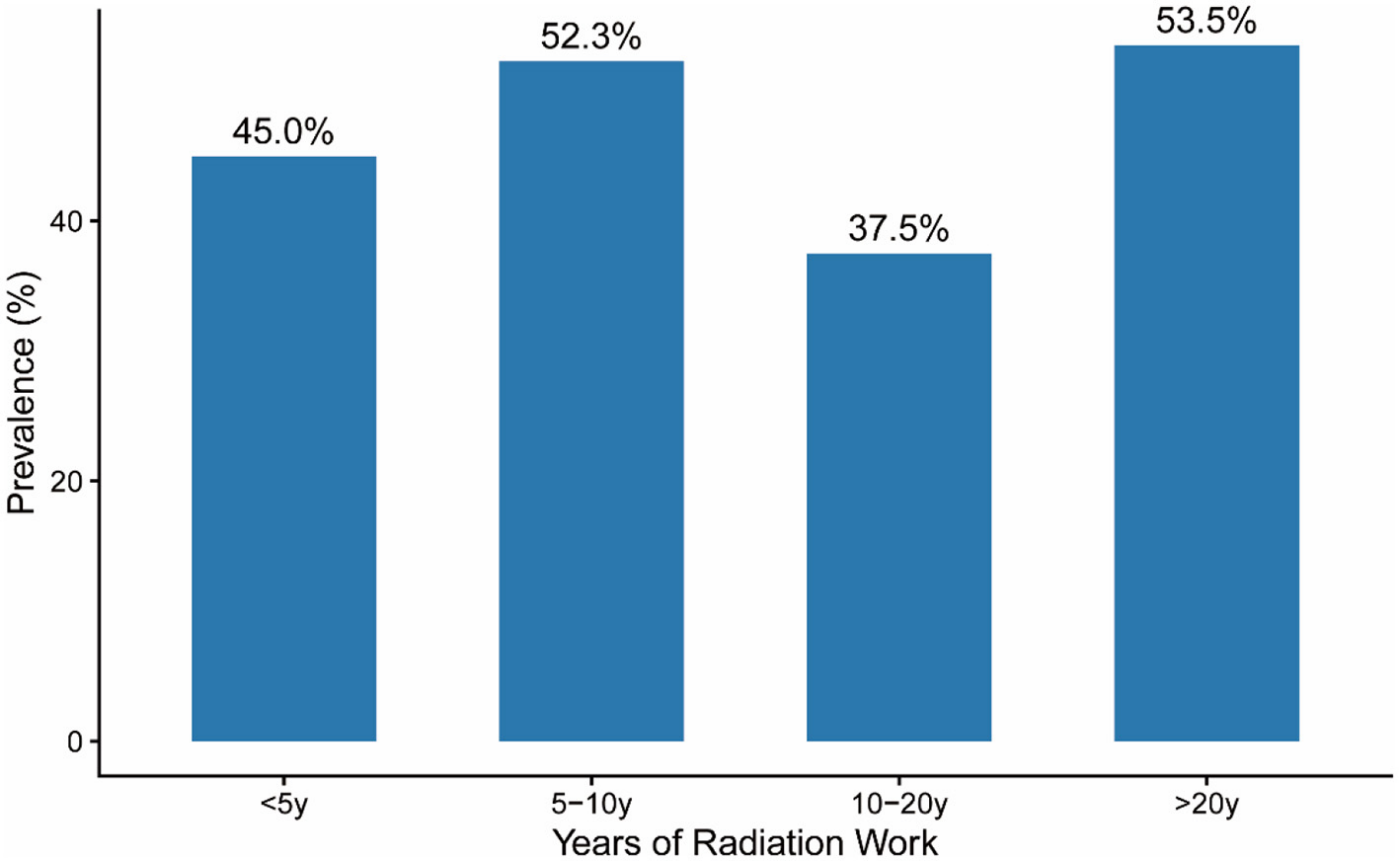
Multiphasic distribution of NAFLD prevalence across occupational tenure groups. The bar chart illustrates the prevalence of Non-Alcoholic Fatty Liver Disease (NAFLD) stratified by years of radiation work. The distribution exhibits an “N-shaped” trajectory, characterized by a peak in the 5–10 year cohort followed by a significant nadir in the 10–20 year group, suggestive of the Healthy Worker Survivor Effect.

Stratification of NAFLD prevalence by duration of radiation work revealed a non-linear distribution rather than a simple monotonic increase (Figure 2). Prevalence was 44.1% in personnel with <5 years of service, increased to 61.9% in the 5–10 years group, declined to 32.5% in the 10–20 years group, and then rebounded to 52.3% in those with >20 years of service. This pattern remained consistent with an early-career peak, a mid-career dip, and a later-career rebound.

### 3.3 Decoupling metabolic drivers from occupational duration

**Figure 3:**
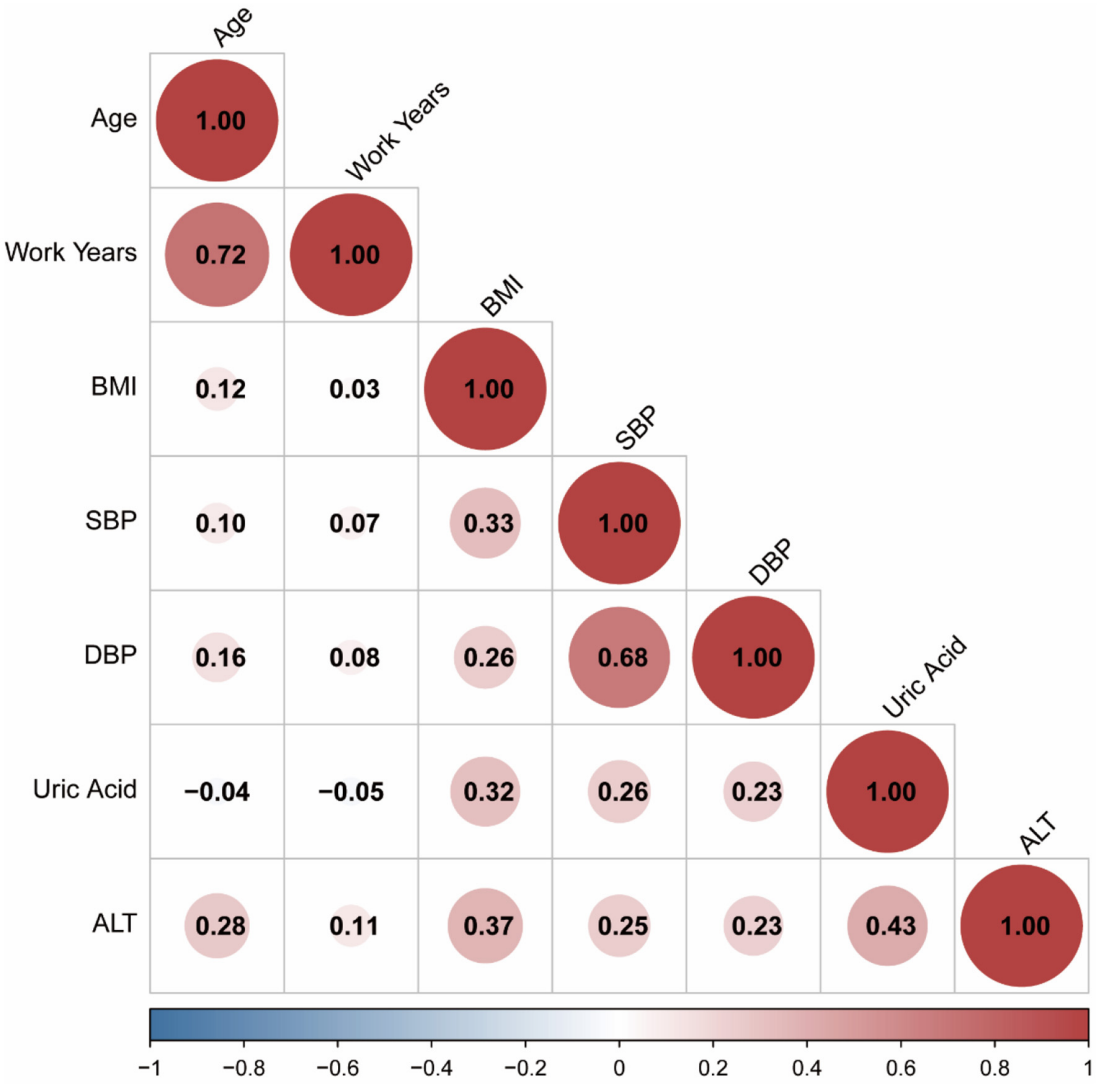
Pearson correlation matrix heatmap of continuous variables. The heatmap visualizes pairwise correlation coefficients (r) between age, occupational tenure (Work Years), and metabolic parameters (BMI, SBP, DBP, Uric Acid, ALT). Color intensity corresponds to the magnitude of the correlation coefficient (scale: −1.0 to 1.0). A strong positive correlation is observed between Age and Work Years, warranting statistical adjustment to prevent multicollinearity in subsequent regression models.

**Figure 4:**
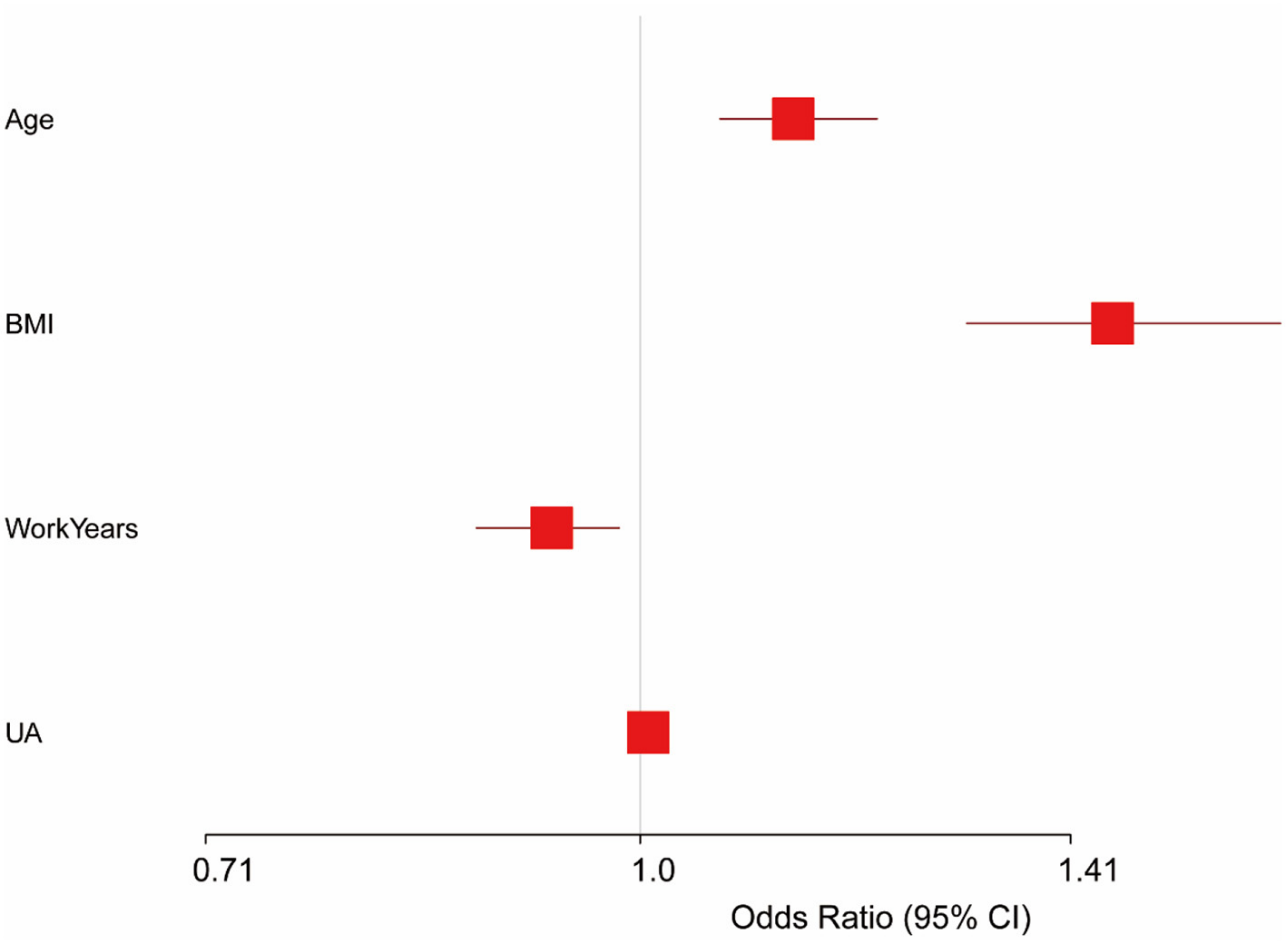
Forest plot of multivariate logistic regression analysis for NAFLD risk factors. The plot displays the adjusted Odds Ratios (OR) and 95% Confidence Intervals (CI) for independent predictors derived from the multivariate model. Body Mass Index (BMI) presents as the dominant risk factor, significantly exceeding unity. Conversely, years of radiation work (Work Years) crosses the reference line (OR ~ 1.0), indicating a lack of independent statistical significance after adjusting for metabolic confounders. Squares represent point estimates; horizontal lines indicate 95% CIs.

**Figure 5:**
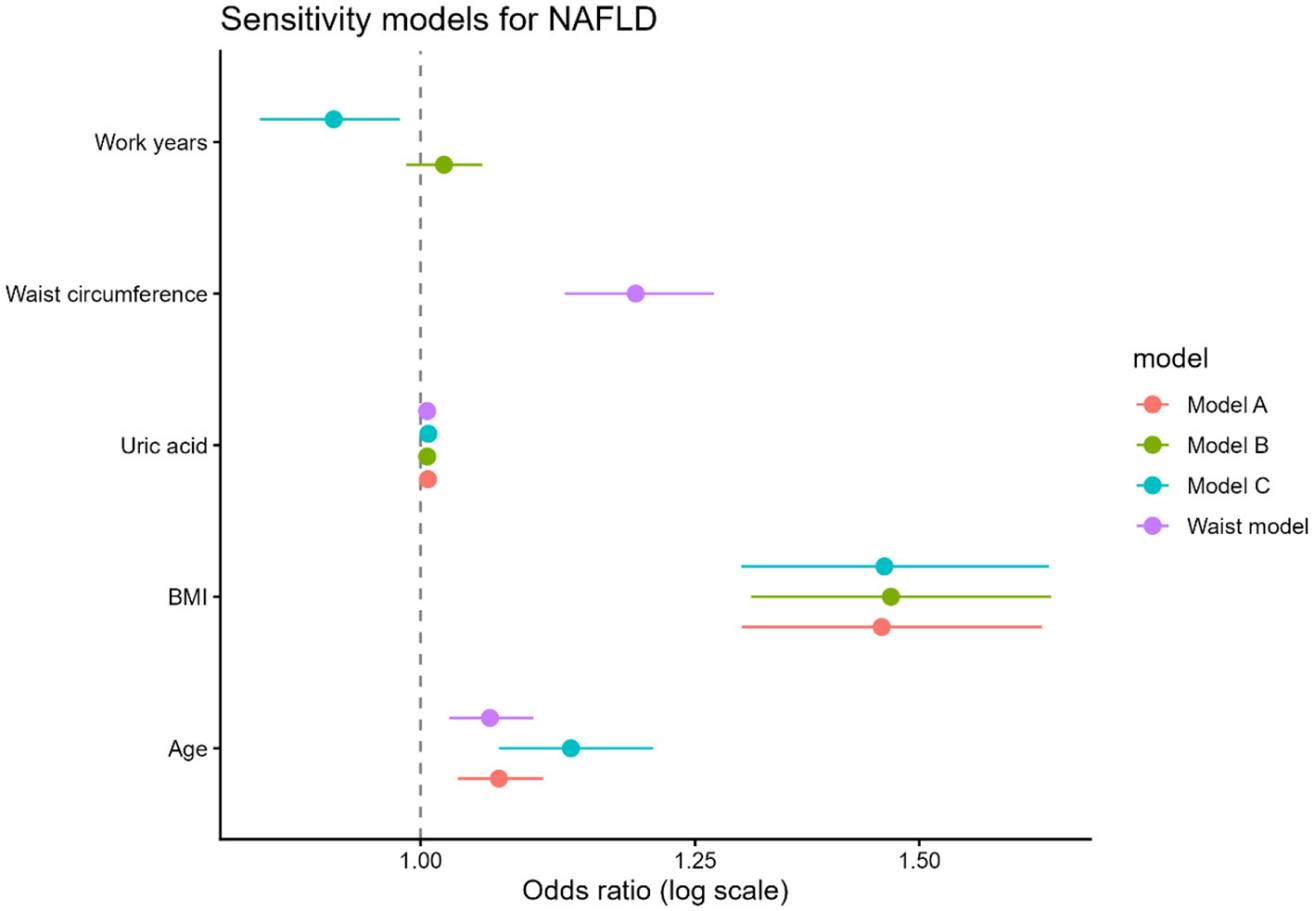
Sensitivity models for NAFLD. The forest plot shows that BMI remained consistently associated with NAFLD across alternative model specifications, whereas the effect of work years was less stable.

To isolate independent predictors of NAFLD and to further evaluate the confounding influence of correlated occupational variables, we conducted additional correlation and multivariable analyses. Age was positively correlated with years of radiation work (r = 0.785), while years of night-shift work also correlated strongly with total work tenure (r = 0.874) (Figure 3), underscoring the challenge of disentangling cumulative schedule burden from occupational duration in cross-sectional data. Formal multicollinearity diagnostics nevertheless showed acceptable VIF values (Age 3.51; Work Years 3.22; BMI 1.08; Uric Acid 1.17; Sex 1.02; Occupation 1.32), indicating that the observed correlation structure did not invalidate model interpretation. In the primary multivariable model, BMI remained associated with NAFLD, whereas the effect of work years was weaker after adjustment (Figure 4). Across supplementary sensitivity models, BMI remained the most stable predictor of NAFLD, with odds ratios ranging from 1.45 to 1.49, whereas work years was not a stable positive predictor across model specifications (Figure 5).

### 3.4 Dose-response trajectory and the survivor effect

**Figure 6:**
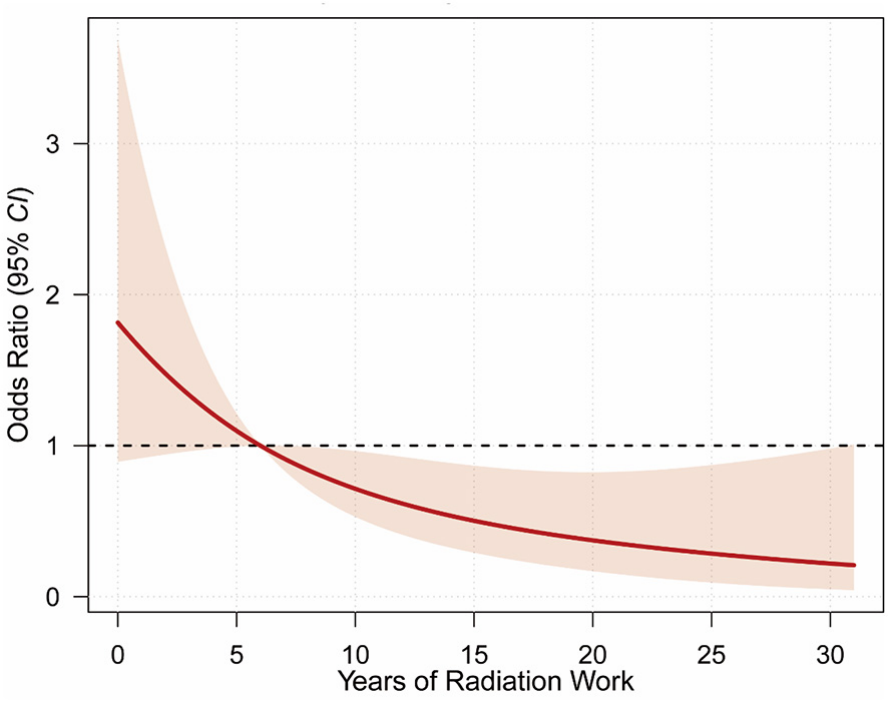
Restricted Cubic Spline (RCS) regression modeling the non-linear association between occupational tenure and NAFLD risk. The solid red line represents the estimated Odds Ratio (OR) for NAFLD across continuous years of radiation work, adjusted for Age, BMI, and Uric Acid. The shaded red area indicates the 95% Confidence Interval. The model utilizes three knots at the 10th, 50th, and 90th percentiles. The trajectory demonstrates a non-monotonic risk profile, with the OR dropping below the reference line (dashed line, OR = 1.0) after approximately 8 years, providing statistical evidence for the survivor bias during the mid-career phase.

**Figure 7:**
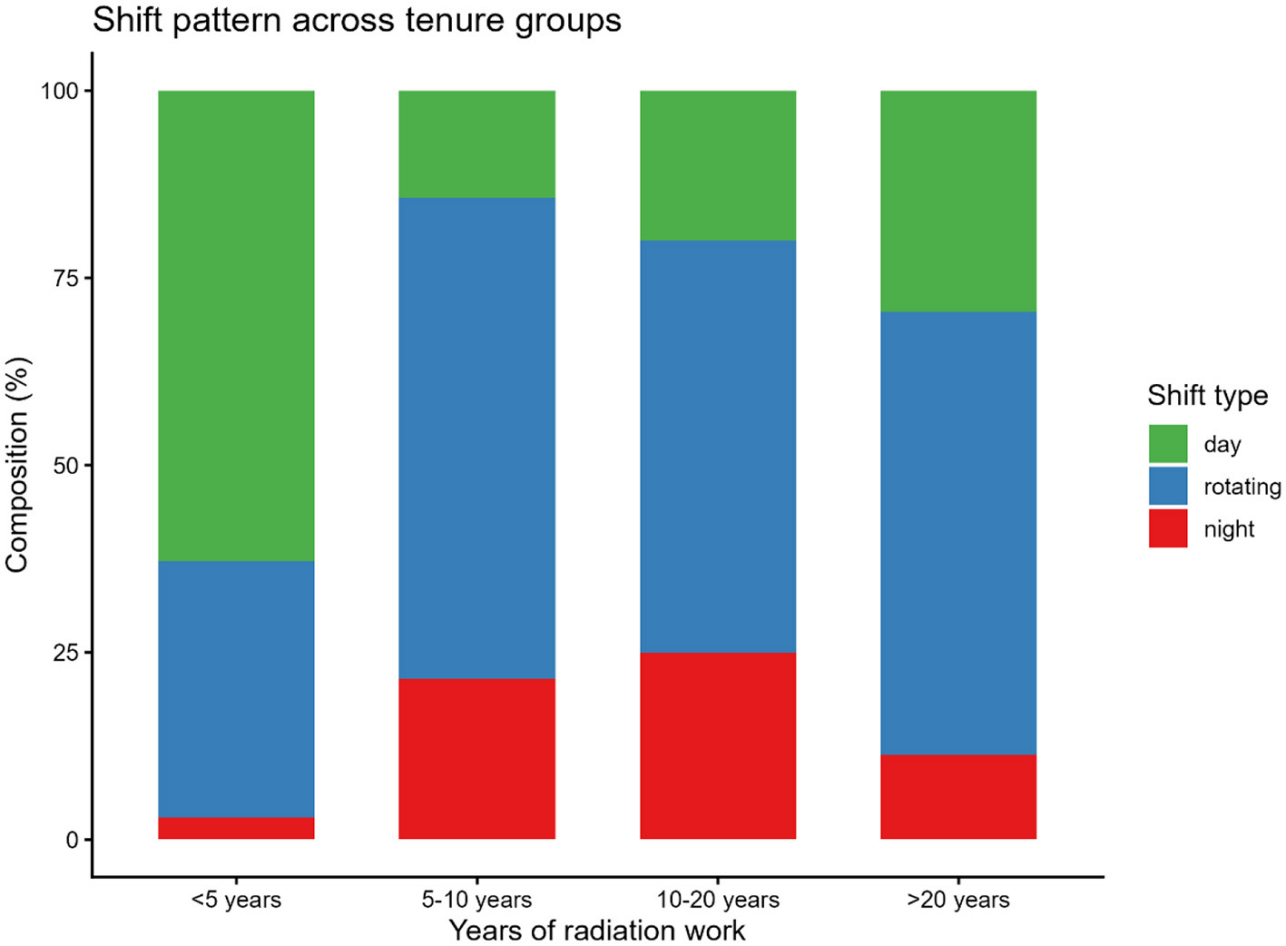
Shift pattern across tenure groups. The stacked bars summarize the composition of day, rotating, and night-shift schedules across tenure strata, illustrating greater rotating/night-shift representation in earlier career stages.

Restricted Cubic Spline (RCS) regression was used to explore the non-linear association between occupational tenure and NAFLD risk after adjustment for relevant covariates (Figure 6). The observed trajectory was compatible with a possible survivor-selection pattern during the mid-career period, but this interpretation should be regarded as hypothesis-generating rather than confirmatory in the absence of longitudinal follow-up. Descriptive schedule analyses further indicated a greater night-shift burden in the 5–10-year group than in the 10–20-year group, with median monthly night shifts of 5.0 (3.0, 7.0) vs. 3.0 (2.0, 5.0), respectively (Figure 7), providing contextual support for greater early-career occupational strain.

### 3.5 Diagnostic performance of metabolic indicators

**Figure 8:**
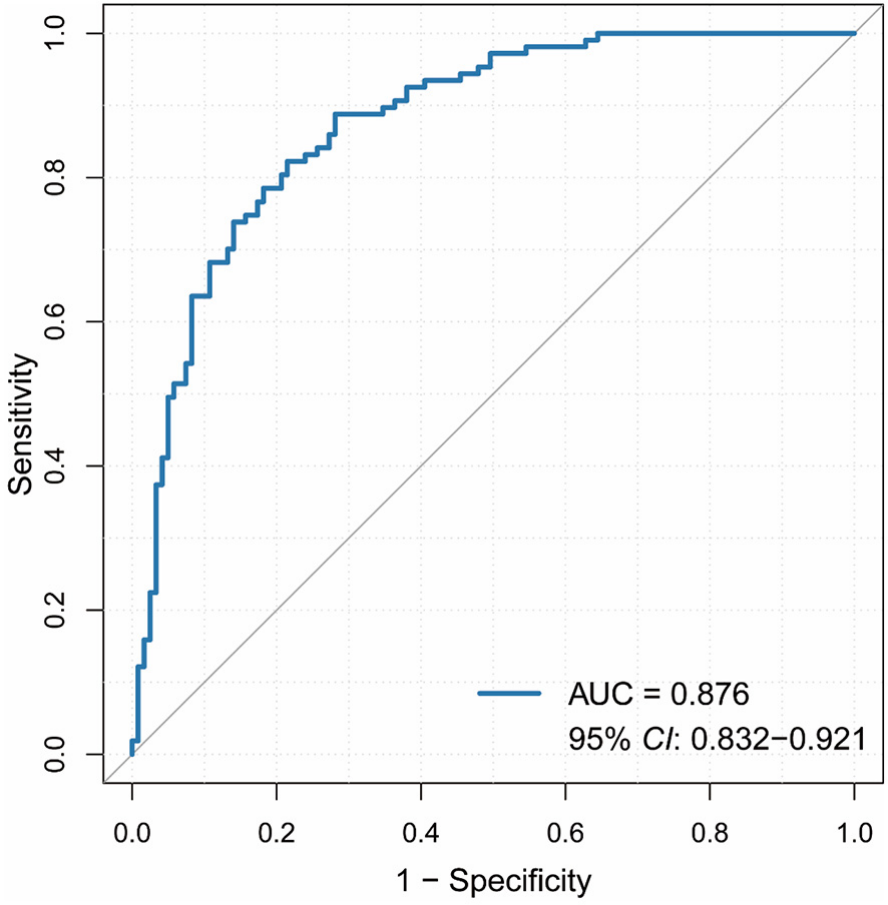
Receiver Operating Characteristic (ROC) curve for the metabolic screening model. The curve evaluates the diagnostic performance of a parsimonious predictive model comprising BMI, Age, and Uric Acid in identifying NAFLD among medical imaging professionals. The model achieves an Area Under the Curve (AUC), indicating high discriminatory accuracy independent of occupational exposure metrics. The diagonal line represents a random classifier.

Receiver Operating Characteristic (ROC) analysis demonstrated robust discriminatory performance for the metabolic screening model comprising BMI, Age, and Uric Acid (Figure 8). The stability of BMI across supplementary sensitivity models further supported the robustness of this parsimonious metabolic screening framework.

## 4 Discussion

The observed NAFLD prevalence of 47.7% in this cohort indicates a substantial metabolic health burden within the medical imaging workforce (12, 13). In this cross-sectional baseline analysis, the most reproducible signal was adiposity-related metabolic risk rather than occupational tenure alone. The elevated burden observed during the early-career period may reflect greater schedule-related strain and metabolic vulnerability. In the present cohort, the 5–10-year group had the highest observed prevalence of NAFLD (61.9%) and also showed a greater night-shift burden than the 10–20-year group at the descriptive level. However, because detailed sedentary behavior and circadian hygiene were not directly quantified using validated instruments in the primary dataset, these mechanisms should be interpreted as plausible contextual explanations rather than measured causal drivers (14). The abrupt transition from training to full-time practice, often involving high-intensity block night shifts, precipitates acute circadian misalignment. Mechanistically, the desynchronization between the central suprachiasmatic nucleus (SCN) and peripheral hepatic oscillators during nocturnal work promotes postprandial hyperglycemia and impairs hepatic lipid clearance (15, 16). This aligns with literature indicating that junior professionals are particularly susceptible to rapid visceral adiposity accumulation and dyslipidemia within their first decade of service, driven by cortisol dysregulation and cumulative sleep debt—a state of chronic “metabolic jetlag” (17).

The reduced prevalence observed in the 10–20-year group (32.5%) may be compatible with a survivor-selection process, but this interpretation cannot be directly confirmed in a cross-sectional study. Rather than constituting proof of a healthy worker survivor effect, the present findings should be viewed as a tenure-related pattern that warrants longitudinal verification and more direct exposure assessment. This phenomenon operates via rigorous selection bias, wherein individuals manifesting severe metabolic intolerance—such as uncontrolled hypertension or fatigue—selectively withdraw from the high-intensity shift-work pool or transfer to non-clinical roles (18). Consequently, the cohort persisting beyond the 10-year mark represents a “survivor” population possessing either innate metabolic resilience or successful coping mechanisms. Similar non-linear risk distributions have been documented in other shift-work industries, confirming that cross-sectional analyses inherently censor the attrition of metabolically susceptible phenotypes, thereby masking the true toxicity of the occupation in mid-career stages.

Beyond tenure-related patterning, the present analyses consistently identified adiposity-related metabolic burden as the more stable explanatory signal. This interpretation was reinforced by supplementary sensitivity analyses, in which BMI remained consistently associated with NAFLD across all model specifications (OR 1.45–1.49). Waist circumference also remained independently associated with NAFLD in the supplementary waist-based model, further supporting the importance of central adiposity in the observed metabolic burden. This finding is consistent with large-scale longitudinal cohorts demonstrating that, after adjustment for lifestyle factors, the correlation between low-dose ionizing radiation and metabolic syndrome lacks statistical significance (ERR = −0.04) (19, 20). While historical associations between radiation and dyslipidemia fueled hypotheses of radiation-induced inflammation, our data suggest these are likely confounding artifacts of the sedentary, high-stress lifestyle inherent to high-volume radiology (21). The magnitude of risk associated with the “J-shaped” BMI curve (HR > 1.5–3.0) substantially exceeds any theoretical toxicity from low-dose radiation (HR ~1.05) (22). Thus, the persistence of surveillance protocols fixated on radiation dosimetry, at the expense of metabolic monitoring, represents a misalignment of occupational health resources.

However, these findings derive from a regional hospital network and should therefore be generalized cautiously, particularly across healthcare systems with different staffing structures, workload patterns, and occupational surveillance practices.

The evolving nomenclature from NAFLD to MASLD underscores the necessity of integrating metabolic risk factors into occupational health paradigms (23, 24). These findings should not be interpreted as diminishing the importance of conventional radiation protection principles such as ALARA. Instead, they suggest that metabolic surveillance may usefully complement existing dosimetry-centered occupational monitoring, particularly for early-career imaging staff who may also experience schedule-related strain. Interventions should be prioritized for early-career professionals (0–10 years), as this period represents the window of highest vulnerability and maladaptation. Rigorous monitoring of BMI, lipid profiles, and circadian hygiene in junior staff is projected to yield greater occupational health dividends than continued hyper-vigilance regarding effectively controlled radiation exposure.

Several limitations should be acknowledged. First, the cross-sectional design precludes causal inference and does not allow direct verification of a healthy worker survivor effect. Second, detailed quantitative measures of sedentary behavior and circadian hygiene were not directly collected using validated instruments. Third, age, occupational tenure, and years of night-shift work were correlated, although VIF diagnostics and sensitivity analyses supported the stability of the adiposity-related findings. Fourth, the study was conducted within a regional hospital network, which may limit broader generalizability. Finally, although missingness in the supplementary shift-related variables was low, it was not zero.

## 5 Conclusion

These findings do not support a simple linear interpretation of tenure-related metabolic risk. Instead, they suggest that adiposity-related metabolic burden is more robustly associated with NAFLD than occupational tenure alone, and that the observed tenure pattern may be compatible with survivor- selection processes. Future studies should directly quantify shift- related, circadian, and behavioral exposures, and metabolic surveillance may usefully complement existing dosimetry-centered occupational monitoring.

## Data Availability

The raw data supporting the conclusions of this article will be made available by the authors, without undue reservation.
